# Mental health mediates the association between cardiorespiratory fitness and academic performance in European schoolchildren

**DOI:** 10.1016/j.jped.2024.10.013

**Published:** 2025-05-14

**Authors:** Adrià Muntaner-Mas, Pedro L. Valenzuela, Tania Pinto-Escalona, Kirk I. Erickson, Óscar Martínez-de-Quel

**Affiliations:** a University of Balearic Islands, Faculty of Education, GICAFE "Physical Activity and Exercise Sciences Research Group", Palma, Spain; b University of the Balearic Islands, Institute of Research and Innovation in Education, Department of Pedagogy and Specific Didactics, Palma, Spain; c GENUD Toledo Research Group, Faculty of Sport Sciences, University of Castilla-La Mancha, Toledo, Spain; d University of Alcalá, Department of Systems Biology, Madrid, Spain; e Complutense University of Madrid, Faculty of Education, Madrid, Spain; f AdventHealth Research Institute, Orlando, FL, United States of America; g University of Pittsburgh, Department of Psychology, Pittsburgh, PA, United States of America; h International University of La Rioja, Faculty of Education, Department in Didactics of Physical Education and Health, Logroño, Spain; i Technical University of Madrid, Faculty of Physical Activity and Sports Sciences-INEF, Madrid, Spain

**Keywords:** Physical fitness, Psychosocial problems, Strengths and difficulties questionnaire, Children, Academic achievement

## Abstract

**Objective:**

The objective of this study was to assess the potential mediating role of mental health in the association between cardiorespiratory fitness (CRF) and academic performance in European schoolchildren.

**Method:**

The study followed a cross-sectional design. 507 schoolchildren (51.5 % girls, 7.4 ± 0.4 years) from 20 schools in five European countries were included in the analyses. Academic performance was assessed using school grades, mental health was assessed through the Strengths and Difficulties Questionnaire (SDQ) for parents, and CRF was estimated through the multistage 20-m shuttle run test. Linear regression and mediation analyses were conducted to test these hypotheses.

**Results:**

Mental health difficulties were associated with worse performance on academic indicators (β ranging from -0.121 to -0.324, *p* < 0.05). Further, mental health difficulties were associated with lower CRF (β ranging from -0.121 to -0.189, *p* < 0.05). Mediation analyses revealed that the association between CRF and academic performance indicators was partially mediated (from 8 % to 25 %) by mental health [except for conduct and peer problems (β ranging from -0.025 to -0.080, *p* > 0.05).

**Conclusion:**

The present results highlight that mental health is a possible mediator in the association between CRF and academic performance. These findings might support the importance of improving CRF levels to reduce mental health difficulties with subsequent potential benefits on academic performance.

## Introduction

Mental disorders account for a large health burden among children in high-income countries.^1^ Children’s mental health (e.g., self-concept, self-esteem, negative and positive affect) is associated with the odds of externalizing or internalizing behaviors (e.g., conduct problems, rule-breaking behavior, attention-deficit/hyperactivity disorder [ADHD]) that can subsequently trigger preclinical psychological states or clinically diagnosed disorders such as depression or anxiety.^2^

In this regard, current evidence suggests that physical activity interventions can improve youth’s mental health.^3^ Physical activity may positively impact mental health via physical fitness, which partially mirrors the dose of physical activity that has occurred over a certain time. Cardiorespiratory fitness (CRF), one of the main physical fitness components, has been recognized as an important health marker throughout the lifespan.^4^ Specifically, higher CRF has been associated with better mental health, as supported by a recent systematic review and meta-analysis that found a relation between higher CRF and lower depressive symptoms in children.^5^ Likewise, a recent meta-analysis on the topic observed small-to-medium-sized associations between youths’ CRF and their self-esteem, self-concept, physical self-perceptions, and well-being.^6^ However, to the best of our knowledge, only Åvitsland et al.^7^ have used a broad measure of mental health (e.g., Strengths and Difficulties Questionnaire [SDQ]) to study its relation with this physical fitness component. There is also strong evidence supporting that CRF is positively associated with cognitive function, and particularly academic performance, in children and adolescents.^8^ Additionally, recent evidence suggests that youth with higher CRF levels present better mental health. On the contrary, mental health problems have been found to have negative consequences on academic performance.^9^ A previous systematic review and meta-analysis support an inverse association between mental health-related outcomes such as poor self-concept, anxiety, or ADHD and academic performance.^10^ Notwithstanding, the hypothesis that impaired mental health could have detrimental effects on academic performance has been mostly investigated through the analysis of specific components (e.g., anxiety, depression), with scarce research analyzing mental health as a broad concept comprising both internalizing and externalizing behaviors.^11^

In this scenario, it is worth noting that little evidence exists on factors that could potentially mediate the purported association between CRF and academic performance. Some of the explanations provided for this positive association are based on physical health indicators or metabolic biomarkers.^12^^,^^13^ However, whether mental health mediates the association between CRF and academic performance remains to be elucidated. To our knowledge, only two studies have studied the role of mental health in the relationship between CRF and academic performance. Xiang et al.^14^ examined this association among 144 Chinese adolescents. The authors found a significant indirect effect (mediation) of overall physical fitness on academic performance (three academic indicators) through depression. Some years later, Monzonís-Carda et al.^15^ investigated whether the risk of depression mediated the association between CRF and academic performance in 263 Spanish adolescents. Their results indicated a significant mediating effect of the risk of depression in the association between CRF and academic performance. However, these studies only included adolescents from a specific country and assessed a limited number of academic performance indicators. Additionally, both studies considered the risk of depression (internalizing behavior) as a mediator, which is a narrower aspect of mental health.

The present hypothesis posits that mental health serves as a mediator in the relationship between CRF and academic performance. Previous research has demonstrated that CRF positively influences mental health by alleviating symptoms of anxiety, depression, and stress, while simultaneously enhancing overall academic performance. Improved mental health is further associated with enhanced academic outcomes. By proposing mental health as a mediating factor, the authors aim to elucidate how CRF may indirectly contribute to better academic performance through its beneficial effects on mental health. Thus, the authors contend that CRF not only directly affects academic performance but also exerts a significant indirect influence via its positive impact on mental health. To address these gaps in the literature, this study aimed to analyze the mediating role of mental health (as a broad concept) in the association between CRF and academic performance in a large sample of European schoolchildren.

## Method

### Study design

The present study had a cross-sectional design and is a secondary analysis of a recent multi-country cluster randomized controlled trial, which details are available elsewhere.^16^ For this study, baseline data of children from both the intervention and control groups were included. Specifically, mental health and CRF were assessed at the beginning of the 2017–18 academic year, while academic performance was determined by examining grades at the end of the previous academic year.

### Participants

The inclusion and exclusion criteria for the study have been previously described.^16^ In brief, the current study included second-grade students (7–8 years) from 40 classrooms across 20 schools (two classrooms per school and four schools per country) from five different European countries (Spain, Portugal, France, Germany, and Poland). In March 2015, the National Karate Federations of the participating countries advertised the project on their websites, inviting schools to participate. Each country selected four schools representing diverse student populations based on geographic location and socioeconomic status, including both public/state and private institutions. The schools included both state and private institutions from participating countries. In June 2017, two second-grade classes with similar characteristics were chosen from each school, and these were randomly assigned to either "control" or "intervention" groups (albeit only baseline data were used in the present study). Of the 721 participants initially enrolled in the multi-country cluster randomized controlled trial, 507 children had valid baseline data for sociodemographic variables, 476 for CRF, and 533 for psychosocial functioning and academic performance.

Online written informed assent was obtained from parents or legal guardians before participating in the investigation. The research procedures were conducted under the Declaration of Helsinki and its later amendments and were approved by the Padova University and Complutense University of Madrid Institutional Review Board for the protection of human subjects.

### Measures

Mental health was measured with SQD, which is a well-known measuring instrument widely used for the assessment of mental health in children and adolescents. A parent/guardian of each participant was asked to rate psychosocial functioning using an online version of the SDQ for parents.^17^ The SDQ is a 25-item screening questionnaire with 5 scales, each consisting of 5 items, generating scores (ranging from 0 to 10) for emotional symptoms, conduct problems, hyperactivity/inattention, peer problems, and prosocial behavior. The first 4 problem scales were summed to generate a psychosocial difficulties score (ranging from 0 to 40). A higher SDQ score indicates worse psychosocial functioning, that is, more psychosocial difficulties.

CRF was assessed using the multistage 20-m shuttle run test. The starting speed was 8.5 km/h, and it was increased every minute (stage) by 0.5 km/h until the child failed to reach the lines in the required time twice in a row. The last completed stage or half-stage was considered as the child’s result. This test has been proposed as a useful and overall valid method for the estimation of CRF in children.^18^

Academic performance was assessed by the teachers on a 10-point scale (0 indicates the lowest and 10 is the highest performance). The grade point average (GPA, i.e., the average score across all subjects) as well as individual scores for the following subjects were included in the analyses: natural sciences, native language, maths, physical education, and arts.

Apart from the above-mentioned variables, the authors also assessed demographic variables including age, sex, height, weight, body mass index (BMI), and socioeconomic status for their analysis as potential confounders. Weight, height, and BMI were measured using standard procedures, and age- and sex-specific BMI cut-offs of the World Obesity Federation were used to determine overweight/obesity.^19^ To assess socioeconomic status, parents answered the Q1009 question from the Short Questionnaire Rotation A (SQR-A) belonging to the World Health Organization. For statistical analysis, socioeconomic status was categorized as low, medium and high.

### Statistical analyses

Descriptive characteristics of the sample are shown as mean and standard deviation (SD) or percentages. The Kolmogorov-Smirnov test and histograms were used to check for normality, and data were log-transformed before statistical analysis when required. Included and excluded participants did not significantly differ in all study variables (all *p* > 0.1). The authors tested sex, age, BMI and socioeconomic status as potential confounders, but analyses were only adjusted for sex and socioeconomic status as sensitivity analyses showed no significant interactions of age, or BMI with psychosocial functioning or CRF (i.e., sex* psychosocial functioning; age* psychosocial functioning; BMI* psychosocial functioning) to academic performance (all *p* > 0.1). Multilevel analysis models were conducted with the Restricted Maximum Likelihood Estimation Method (Logarithm of Likelihood −2). Neither the “school” nor the “country” factors showed statistically significant effects (*p* > 0.05), and therefore analyses were not adjusted for clustering at the school or the country level.

Linear regression analyses were used to study the association of CRF (independent variable) with mental health and academic performance, and the association of mental health (independent variable) with academic performance. The authors corrected for multiple testing by defining statistical significance using a Benjamini-Hochberg False Discovery Rate of *Q* < 0.10.^20^ Mediation analyses were performed using the PROCESS macro according to the procedures proposed by Hayes. In these models, academic performance was the dependent variable, CRF the independent and mental health indicators the mediators. Accordingly, total (c) and direct effects (a, b, c’) were computed as indicated by the unstandardized regression coefficient between the variables in each model, and the indirect effect was calculated as the product of the a*b coefficient. Mediation analyses were considered significant when zero was not included in the 95 % confidence interval of the indirect effects, estimated by 10,000 bootstrap samples. Additionally, the authors tested mediation according to Sobel by dividing the indirect effect by its standard error and performing a z-test. The part of the total effect that was explained by the mediation, namely the percentage of mediation, was calculated as follows: (indirect effect/total effect) x 100. Analyses were performed using SPSS (v23.0; IBM Corp, Armonk, NY) and the level of significance was set at *p* < 0.05.

## Results

Descriptive characteristics of the study sample are presented in Table 1. Table 2 shows the association between CRF, mental health indicators and academic performance, adjusted for confounders. Specifically, mental health difficulties were associated with poorer academic performance indicators (β ranging from −0.121 to −0.325, *p* < 0.05) and lower CRF (β ranging from −0.121 to −0.189, *p* < 0.05), except for conduct problems. Further, as predicted, higher CRF was associated with higher academic performance indicators (β ranging from 0.249 to 0.337, *p* < 0.05).

**Table 1:** Characteristics of the study participants.

	All		Boys		Girls	
	Mean or n	SD or %	Mean or n	SD or %	Mean or n	SD or %
						
*Sociodemographic*						
Age (years)	7.42	0.41	7.46	0.44	7.37	0.37
Sex (n, %)	–		–		261	51.5
Socioeconomic status (n, %)						
Low	58	11.5	24	9.8	34	13
Medium	246	48.5	126	51.2	120	46
High	203	40	96	39	107	41
Height (m)	1.26	0.06	1.27	0.06	1.26	0.06
Weight (kg)	26.94	5.28	27.23	5.13	26.66	5.42
Body mass index (kg/m^2^)	16.81	2.30	16.87	2.30	16.74	2.31
Overweight / Obese (n, %)^a^	122	24.1	54	22	68	26.1
*Cardiorespiratory fitness (stages)*	2.87	1.47	2.95	1.54	2.78	1.40
*Academic performance (0–10)*						
GPA	7.81	1.44	7.68	1.39	7.96	1.49
Natural sciences	7.93	1.80	7.83	1.79	8.04	1.82
Native language	7.39	1.96	7.21	1.98	7.58	1.93
Maths	7.66	1.89	7.72	1.80	7.58	1.99
Physical education	7.81	1.59	7.51	1.59	8.13	1.53
Arts	7.66	1.50	7.66	1.49	7.67	1.51
b*Mental health*						
Psychosocial difficulties (0–40)	18.30	5.05	18.65	5.07	17.93	5.02
Emotional problems (0–10)	2.24	1.90	2.24	1.92	2.23	1.89
Conduct problems (0–10)	1.87	1.59	1.95	1.56	1.79	1.63
Hyperactivity/inattention (0–10)	4.27	2.53	4.62	2.52	3.90	2.50
Peer problems (0–10)	1.59	1.63	1.69	1.67	1.48	1.59

Values are expressed as means ± standard deviations unless otherwise indicated.

a Classified according to the World Obesity Federation cut-off points.

b Lower scores indicate better psychosocial functioning. GPA, grade point average. The valid sample for sociodemographic variables was 507, for cardiorespiratory fitness 476, and for academic performance and mental health domains 533.

**Table 2:** Linear regression analyses between cardiorespiratory fitness, mental health and academic performance in children.

Mental health					
	Psychosocial difficulties	Emotional problems	Conduct problems	Hyperactivity/inattention	Peer problems
	β (95 % CI)	β (95 % CI)	β (95 % CI)	β (95 % CI)	β (95 % CI)
					
Cardiorespiratory fitness (stages)	**−0.189** (−1.00, −0.28)^b^	**−0.153** (−0.33, −0.05)^a^	−0.076 (−0.20, −0.03)	**−0.145** (−0.40, −0.06)^a^	**−0.121** (−0.24, −0.01)^a^
Grade point average (0–10)	**−0.314** (−1.09, −0.06)^b^	**−0.170** (−0.18, −0.06)^b^	**−0.187** (−0.24, −0.09)^b^	**−0.246** (−0.24, −0.14)^b^	**−0.150** (−0.20, −0.05)^b^
Natural sciences (0–10)	**−0.228** (−0.10 −0.04)^b^	**−0.133** (−0.19, −0.03)^a^	**−0.144** (−0.25, −0.06)^a^	**−0.230** (−0.21, −0.09)^b^	**−0.146** (−0.24, −0.05)^a^
Native language (0–10)	**−0.287** (−0.13 −0.07)^b^	**−0.158** (−0.24, −0.06)^b^	**−0.185** (−0.32, −0.11)^b^	**−0.325** (−0.30, −0.18)^b^	**−0.136** (−0.25, −0.05)^a^
Maths (0–10)	**−0.255** (−0.12 −0.06)^b^	**−0.142** (−0.21, −0.05)^a^	**−0.133** (−0.25, −0.05)^a^	**−0.302** (−0.28, −0.15)^b^	**−0.124** (−0.23, −0.04)^a^
Physical Education (0–10)	**−0.315** (−0.12, −0.06)^b^	**−0.226** (−0.24, −0.10)^b^	**−0.179** (−0.26, −0.08)^b^	**−0.301** (−0.23, −0.12)^b^	**−0.198** (−0.26, −0.10)^a^
Arts (0–10)	**−0.315** (−0.12, −0.07)^b^	**−0.109** (−0.16, −0.01)^a^	**−0.136** (−0.22, −0.05)^a^	**−0.324** (−0.26, −0.15)^b^	**−0.121** (−0.20, −0.03)^a^

β = Standardized coefficient. Statistically significant values are shown in bold.^a^*p* < 0.05.^b^*p* < 0.001. The bold font indicates that the specific association surpassed the Benjamini-Hochberg correction for multiple comparison tests (performed for each academic performance domain).

In the relationship between cardiorespiratory fitness and mental health domains, the former was introduced as the independent variable. In the relationship between mental health and academic performance, the former was introduced as an independent variable. Each exposure variable was analyzed in a separate regression model for each outcome variable.

CI: confidence interval. The analyses were adjusted by sex and socioeconomic status.

The valid sample for the cardiorespiratory fitness model was 342 and 466 for the academic performance indicators.

**Figure 1:**
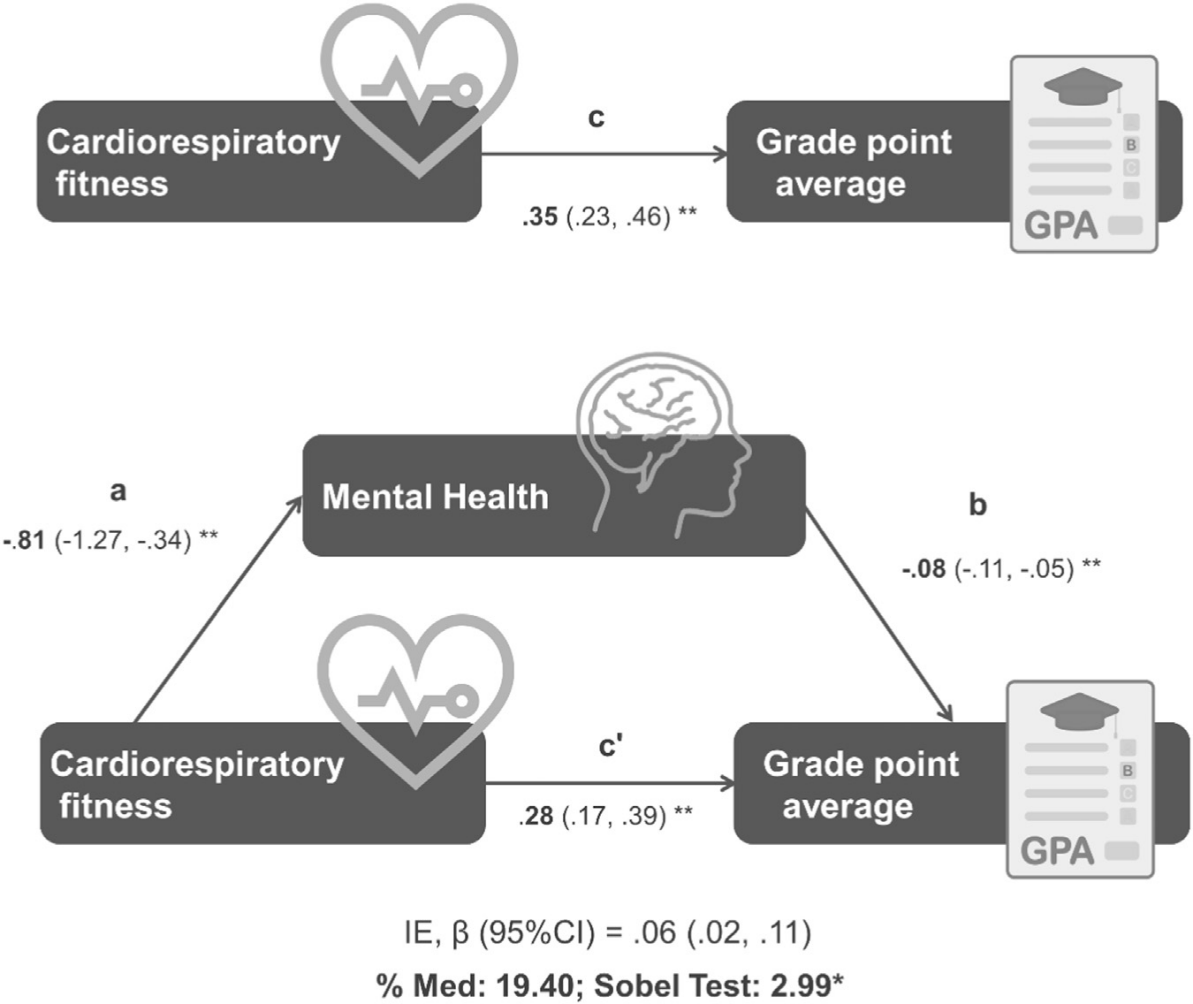
Mental health mediation model of the relationship between cardiorespiratory fitness with grade point average. Results are shown as regression coefficients along with 95 % confidence interval. Statistically significant values are shown in bold. p-value = ^a^*p* < 0.01 and *p < 0.05. IE (95 %CI), Indirect Effect (95 % Confidence Interval): %Med, percentage mediated by the proposed mediator. Path a, effect of X on M; path b, effect of M on Y; path c', direct effect of X on Y controlling for M; path a*b, indirect effect of X on Y through M; path c, total effect of X on Y (direct and indirect). X, predictor variable; Y, outcome variable; M, mediator. In these models, Y was academic performance, M was mental health and X was cardiorespiratory fitness. The analyses were adjusted by sex and socioeconomic status. The valid sample was 288.

The results from the mediation analyses are shown in Figure 1 and Table 3. Overall, CRF was inversely associated with mental health difficulties [except for conduct problems, (path a)]. In addition, mental health difficulties were negatively associated with all academic performance indicators [except for conduct and peer problems, (path b)]. A significant total effect (path c) was observed for CRF with all academic performance indicators. The direct effect (path c’) of CRF on academic performance indicators when mental health was included in the model was significant for all academic performance indicators (except for some mental health indicators). Mediation analyses (path a*b) revealed that the association between CRF and academic performance indicators was partially mediated via mental health difficulties (except for conduct and peer problems), and this mediation ranged from 8 % to 25 %, (β ranging, from −0.025 to −0.080, *p* > 0.05).

**Table 3:** Total, direct, and indirect effects of the mediation analyses investigating mental health as a mediator between cardiorespiratory fitness and academic performance, controlling for sex and socioeconomic level.

Outcomes	Mediators	Path a	Path b	DE (path c’)	TE (path c)	IE a*b β (95 % CI)	P_M (%)_
GPA	Psychosocial difficulties	**−0.812^a^**	**−0.083**	**0.283^a^**	**0.351^a^**	**0.067 (0.02, 0.11)**	19.4
	Emotional problems	**−0.256^b^**	**−0.122^b^**	**0.319^a^**	**0.351^a^**	**0.031 (0.00, 0.06)**	9.1
	Conduct problems	−0.121	−0.131	**0.335^a^**	**0.351^a^**	0.016 (−0.00, 0.04)	–
	Hyperactivity/inattention	**−0.313^b^**	**−0.199^a^**	**0.288^a^**	**0.351^a^**	**0.062 (0.01, 0.12)**	17.9
	Peer problems	**−0.230^b^**	−0.074	**0.333^a^**	**0.351^a^**	0.017 (−0.00, 0.04)	–
							
Natural sciences	Psychosocial difficulties	**−0.812^a^**	**−0.087^a^**	**0.251^a^**	**0.322^a^**	**0.070 (0.02, 0.12)**	22
	Emotional problems	**−0.256^b^**	**−0.122^b^**	**0.290^a^**	**0.322^a^**	**0.031 (0.00, 0.07)**	9.9
	Conduct problems	−0.121	−0.108	**0.309^a^**	**0.322^a^**	0.013 (−0.00, 0.04)	–
	Hyperactivity/inattention	**−0.313^b^**	**−0.196^a^**	**0.260^a^**	**0.322^a^**	**0.061 (0.01, 0.12)**	19.3
	Peer problems	**−0.230^b^**	−0.102	**0.298^a^**	**0.322^a^**	0.014 (−0.01, 0.05)	–
							
Native Language	Psychosocial difficulties	**−0.812^a^**	**−0.097^a^**	**0.362^a^**	**0.441^a^**	**0.078 (0.03, 0.14)**	17.9
	Emotional problems	**−0.256^b^**	**−0.141^b^**	**0.404^a^**	**0.441^a^**	**0.036 (0.01, 0.00)**	8.4
	Conduct problems	−0.121	−0.158	**0.421^a^**	**0.441^a^**	0.019 (−0.00, 0.05)	–
	Hyperactivity/inattention	**−0.313^b^**	**−0.240^a^**	**0.365^a^**	**0.441^a^**	**0.075 (0.01, 0.15)**	17.2
	Peer problems	**−0.230^b^**	−0.061	**0.427^a^**	**0.441^a^**	0.014 (−0.01, 0.05)	–
							
Maths	Psychosocial difficulties	**−0.812^a^**	**−0.083^a^**	**0.347^a^**	**0.415^a^**	**0.067 (0.02, 0.12)**	16.4
	Emotional problems	**−0.256^b^**	**−0.139^b^**	**0.379^a^**	**0.415^a^**	**0.035 (0.01, 0.07)**	8.7
	Conduct problems	−0.121	**−0.208^a^**	**0.292^a^**	**0.415^a^**	0.009 (−0.00, 0.03)	–
	Hyperactivity/inattention	**−0.313^b^**	**−0.228^a^**	**0.343^a^**	**0.415^a^**	**0.071 (0.01, 0.13)**	17.1
	Peer problems	**−0.230^b^**	−0.027	**0.408^a^**	**0.415^a^**	0.006 (−0.02, 0.04)	–
							
Physical Education	Psychosocial difficulties	**−0.812^a^**	**−0.099^a^**	**0.237^a^**	**0.317^a^**	**0.080 (0.03, 0.13)**	25.2
	Emotional problems	**−0.256^b^**	**−0.178^b^**	**0.271^a^**	**0.317^a^**	**0.045 (0.01, 0.08)**	14.5
	Conduct problems	−0.121	**−0.208^a^**	**0.292^a^**	**0.317^a^**	0.025 (−0.00, 0.06)	–
	Hyperactivity/inattention	**−0.313^b^**	**−0.195^a^**	**0.256^a^**	**0.317^a^**	**0.061 (0.01, 0.12)**	19.2
	Peer problems	**−0.230^b^**	**−0.111^b^**	**0.291^a^**	**0.317^a^**	**0.025 (0.00, 0.06)**	8.2
							
Arts	Psychosocial difficulties	**−0.812^a^**	**−0.085^a^**	**0.279^a^**	**0.349^a^**	**0.069 (0.02, 0.12)**	20.1
	Emotional problems	**−0.256^b^**	−0.085	**0.328^a^**	**0.349^a^**	0.020 (−0.00, 0.05)	–
	Conduct problems	−0.121	**−0.160^b^**	**0.329^a^**	**0.349^a^**	0.019 (−0.00, 0.05)	–
	Hyperactivity/inattention	**−0.313^b^**	**−0.205^a^**	**0.285^a^**	**0.349^a^**	**0.064 (0.01, 0.13)**	18.3
	Peer problems	**−0.230^b^**	−0.053	**0.337^a^**	**0.349^a^**	0.012 (−0.01, 0.04)	–

Results are shown as regression coefficients along with 95 % confidence interval. Statistically significant values are shown in bold. p-value = ^a^*p* < 0.01 and ^b^*p* < 0.05.

IE (95 %CI), Indirect Effect (95 % Confidence Interval); P_M (%)_, percentage mediated by the proposed mediator. Path a, effect of X on M; path b, effect of M on Y; path c', direct effect of X on Y controlling for M; path a*b, indirect effect of X on Y through M; path c, total effect of X on Y (DE and IE). X, predictor variable; Y, outcome variable; M, mediator. In these models, Y was academic performance, M was mental health indicators and X was cardiorespiratory fitness.

DE, direct effect; TE, total effect; GPA, grade point average.

The analyses were adjusted by sex and socioeconomic status.

The valid sample was 288.

## Discussion

The main purpose of this study was to analyze the potential mediating role of mental health in the relationship between CRF and academic performance indicators. The authors found that CRF was positively associated with academic performance. Moreover, mental health difficulties were inversely associated with both CRF and academic performance. Indeed, mental health difficulties partially mediated the association between the above-mentioned indicators, which suggests that the benefits of CRF on academic performance are partially due to improved mental health. These results highlight the role of mental health in the association of CRF and academic performance and might support the importance of physical activity interventions for enhancing academic performance in children.

One of the main findings of the present study was that mental health was not only associated with CRF and academic performance but also partially mediated the association between these factors. There is biological evidence to further support this mediation effect. For example, lower grey matter volume has been associated with poorer mental health (i.e., inattention, hyperactivity, disruptive behavior, or callous-unemotional traits) in adolescents,^21^ whereas, as mentioned above, physical activity has been reported to improve neurogenesis and CRF has been positively associated with grey matter volumes in children and adolescents. Therefore, the present results are of potential relevance given that CRF is a modifiable factor that could be a target outcome to be included in interventions aiming to improve academic performance and mental health in children. It is worth noting that a recent study by Monzonís-Carda et al.^15^ found somewhat similar results. However, they evaluated whether the risk of depression moderated the relationship between CRF and academic performance in a Spanish cohort of adolescents (13.9 ± 0.3 years). The main difference between their study and ours is that the authors proposed mental health (as a broad domain) as a possible underlying mechanism in the relationship between CRF and academic performance, whereas they examined the risk of depression as a mechanism. In this context, the authors can speculate that the mediating role of mental health in the relationship between CRF and academic performance can be attributed to a complex interaction of neurobiological, psychosocial, and behavioral mechanisms. For instance, CRF is associated with enhanced brain health, particularly in regions such as the hippocampus and prefrontal cortex, which are essential for learning, memory, and executive function. These neurobiological improvements can positively impact mental health by reducing symptoms of depression and anxiety, thereby supporting cognitive processes crucial for academic tasks.^22^ Furthermore, CRF promotes better sleep quality, which is strongly associated with cognitive performance and learning. Increased motivation and self-efficacy resulting from greater CRF also contribute to better academic outcomes by fostering a positive mental state, which enhances focus and goal-setting abilities. As a result, better mental health, driven by these mechanisms, might lead to improved cognitive function, which is critical to enhance academic performance. In sum, to the best of our knowledge, the present study is the first that assessed the mediation role of mental health on the association between CRF and multiple academic performance indicators in the children population.

In line with previous research, the present study confirmed an association between mental health and academic performance.^11^^,^^23^ Behavioral problems (e.g., conduct problems, peer problems) have been previously associated with impaired performance in activities such as reading or numeracy, thus increasing the risk of academic failure. Indeed, Keilow et al.^24^ reported that both the total score and the subscales of the SDQ were inversely associated with performance in academic tests for reading and maths in sixth-grade students. Recent research has also confirmed an inverse association between SDQ and maths scores^25^ and the risk of depression and academic performance.^26^ There is therefore evidence indicating that children with mental health difficulties are at an increased risk of poor academic performance, although the causal relationship between these variables could be bidirectional.

The present results also show that lower CRF in children is associated with mental health difficulties. Although these findings differ from a previous study in Canadian children that found a non-significant association between CRF and mental health difficulties, the present study is in agreement with previous research in adolescents^27^ and adults.^28^ A relationship between children’s CRF and other mental health indicators has been found in most studies, including depressive symptoms, stress and optimism. Moreover, higher physical activity levels – which are usually related to high CRF – have been associated with improvements in mental health and particularly self-perception.

In line with the above-mentioned findings, there is also strong evidence that supports a positive relationship between CRF and academic performance, as confirmed by a recent systematic review with meta-analysis including 48 studies.^8^ Several mechanisms have been proposed to explain the positive association between CRF and academic performance or cognitive function. Notably, higher CRF has been positively associated with the development of distinctive brain regions implicated in greater academic performance, such as grey matter volumes in frontal regions (i.e., premotor cortex and supplementary motor cortex) and subcortical regions (i.e., hippocampus). Moreover, physical activity and particularly physical exercise – arguably the most effective intervention for the improvement of CRF – have been reported to improve cognitive function, which seems to be at least partly due to the release of brain-derived neurotrophic factor as well as to the promotion of synaptic plasticity and hippocampal neurogenesis.^29^

Some limitations of the present study should be acknowledged, notably its cross-sectional design, which prevents us from concluding a causal relationship. Thus, the statistical mediation results should be interpreted in this context. Furthermore, sampling did not include all European schools but only schools that volunteered to participate in the study, which might induce some bias and might limit generalizability. Another limitation of this study is the varying sample sizes across analyses due to the availability of valid data for each outcome. This may affect the generalizability of the findings and should be considered when interpreting the results. In any case, participating countries represent a wide part of the European population and schools were selected aiming to represent the majority of schoolchildren of each country (location, socioeconomic status, private and public schools). Conversely, some strengths of this study should be highlighted, such as the relatively large sample size, the number of schools, and the multicountry nature.

## Conclusion

The present study shows that mental health might be a possible mediator in the association between CRF and academic performance. Although longitudinal studies are warranted, these findings suggest that enhancing CRF during childhood may contribute to better academic performance, partially via improvements in mental health. The present results highlight the role of mental health in children’s CRF and academic performance and might support the importance of physical activity interventions, particularly in individuals with mental health difficulties and low CRF levels.

## Conflicts of interest

The authors declare no conflicts of interest.
